# G protein–coupled receptor 37 biomarker potential in Parkinson’s disease: Inflammation might be the hidden trigger

**DOI:** 10.4103/NRR.NRR-D-25-00581

**Published:** 2025-09-03

**Authors:** Josep Argerich, Marc López-Cano, Francisco Ciruela

**Affiliations:** Pharmacology Unit, Department of Pathology and Experimental Therapeutics, School of Medicine and Health Sciences, Institute of Neurosciences, University of Barcelona, Barcelona, Spain; Neuropharmacology & Pain Group, Neuroscience Program, Bellvitge Institute for Biomedical Research, L’Hospitalet de Llobregat, Spain

**Figure 1:**
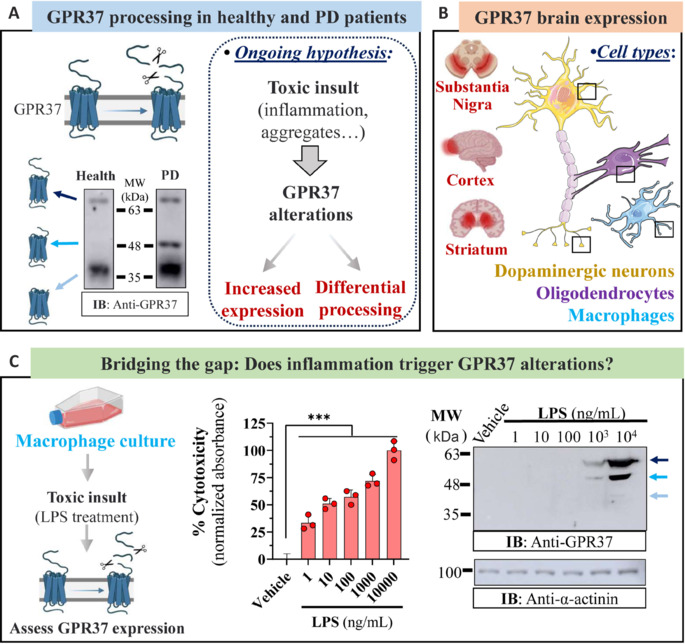
Expression and processing of GPR37 in neurodegeneration conditions and lipopolysaccharide-activated macrophages. (A) GPR37 expression and processing in healthy individuals and PD patients. The scheme illustrates the generation of cleaved GPR37 forms by proteases (i.e., ADAM10; indicated by scissors) and the subsequent release of N-terminal fragments (ecto-GPR37). A representative IB using an antibody targeting the receptor’s C-terminus (anti-GPR37) shows the differential expression of GPR37 in PD striatal necropsies compared to healthy controls (Argerich et al., 2024). The current working hypothesis is illustrated in the right panel. (B) Brain regions assessed for GPR37 expression in neurodegenerative conditions. The scheme highlights human brain areas where GPR37 expression has been examined in the context of neurodegeneration. Expression has been reported in dopaminergic neurons, oligodendrocytes, and macrophages, as indicated by colored cell types. (C) Effect of an inflammatory insult on GPR37 expression. Workflow to study the expression of GPR37 in a murine macrophage cell line (RAW 264.7) treated with the cytotoxic LPS. Graph bar shows the LPS-induced cytotoxicity of RAW 264.7 cells assessed by monitoring the release of lactate dehydrogenase, following manufacturer’s instructions (Product reference: 11644793001; Roche Diagnostics GmbH). Briefly, RAW 264.7 cells (2 × 10^6^ cells/mL) were preincubated with increasing concentrations of LPS for 16 hours at 37°C. Subsequently, the supernatant was collected and lactate dehydrogenase quantified. Data are represented as means ± SD of three independent cultures. ***P < 0.001, *vs*. vehicle, one-way analysis of variance followed by Dunnett’s *post-hoc* test. In the right panel, representative immunoblot showing the expression of GPR37 in RAW264.7 cells following the LPS treatment indicated in the graph bar (left panel). RAW 264.7 cells membrane extracts were analyzed by immunoblot using anti-GPR37 antibodies, as previously described (Argerich et al., 2024). Each GPR37 form is indicated with blue-colored arrows. Created with BioRender.com and Microsoft PowerPoint. GPR37: G protein-coupled receptor 37; IB: immunoblot; LPS: lipopolysaccharide; MW: molecular weight; PD: Parkinson’s disease.

G protein–coupled receptor 37 (GPR37) is an orphan receptor predominantly expressed in the brain, particularly in oligodendrocytes and certain types of neurons. Notably, it has been shown that the N-terminal domain of GPR37 undergoes proteolysis under normal physiological conditions, resulting in the formation of cleaved receptor forms and the release of its ectodomain (ecto-GPR37) into the extracellular milieu (Mattila et al., 2021). Importantly, ecto-GPR37 density is increased in cerebrospinal fluid (CSF) of patients suffering from sporadic Parkinson’s disease (PD), together with an abnormal GPR37 processing in post-mortem PD substantia nigra (Morató et al., 2021; **Figure 1A**). Recently, we demonstrated that GPR37 density upregulation extends to other key brain regions, concretely the prefrontal cortex and striatum (**Figure 1B**), during the early stages of the disease, but not to other neurodegenerative disorders with overlapping symptoms, including corticobasal degeneration, progressive supranuclear palsy, and multiple system atrophy (Argerich et al., 2024). However, the most striking finding was that the increase in GPR37 density was accompanied by elevated levels of CSF ecto-GPR37, observed exclusively in patients with slow-progressing PD. Thus, these results suggest that GPR37 has a differential regulation on PD pathology. Indeed, ecto-GPR37 might serve as a biomarker for predicting disease progression rates (Argerich et al., 2024). In summary, GPR37 has emerged as a potential marker for the post-mortem stratification of neurodegenerative diseases, while ecto-GPR37 shows promise as a predictive biomarker for the progression of PD (Morató et al., 2021; Argerich et al., 2024).

Neurodegenerative diseases are characterized by the gradual and progressive loss of specific neural cell populations. This degeneration is largely associated with the accumulation of misfolded proteins, leading to the formation of abnormal protein aggregates. In PD, α-synuclein aggregates in the soma and neurites of neurons, forming Lewy bodies and Lewy neurites, respectively. These Lewy bodies generally spread from the lower brainstem nuclei to the neocortex, and their presence is closely associated with PD symptomatology. Notably, GPR37 has been found aggregated within the core of Lewy bodies in cases of sporadic PD (Murakami et al., 2004). It remains unclear whether GPR37 is altered because of Lewy body formation or if its dysfunction precedes their formation. Our recent findings suggest that the expression and processing of GPR37 is already impaired in early stages of the disease, in brain regions where α-synuclein aggregates are not typically expected (Argerich et al., 2024). This may suggest that GPR37 dysfunction may precede the formation of α-synuclein aggregates. Furthermore, we and others have shown that GPR37 transcripts are predominantly expressed in mature oligodendrocytes rather than neurons (Argerich et al., 2024), where α-synuclein primarily accumulates in PD. Therefore, it is likely that GPR37 alterations and Lewy body formation occur in different cell types. Given this, it is tempting to speculate that GPR37 dysfunction in oligodendrocytes might trigger early molecular events that go in parallel with PD pathology, being potentially independent of α-synuclein accumulation. In line with this hypothesis, transcriptomic data from PD brain samples have revealed that, in early disease stages, oligodendrocyte-related genes are upregulated in the substantia nigra, while no significant changes are observed in dopaminergic neurons, pointing to a possible causative role for oligodendrocytes in PD (Bryois et al., 2020). This suggests that the observed upregulation of GPR37 in the substantia nigra of PD might originate from oligodendrocytes, though this requires further confirmation. Altogether, we cannot rule out the possibility that GPR37 dysfunction occurs before the appearance of protein aggregates, likely in oligodendrocytes, where the receptor is predominantly expressed.

The disease-stage-dependent expression of GPR37 suggests that the receptor may play a role in early neurodegenerative processes, including impaired autophagy and inflammation. Importantly, recent studies have shown that GPR37 is involved in resolving inflammatory responses (Bang et al., 2018). For instance, macrophages from GPR37 knock-out mice, when exposed to inflammatory stimuli, switched from a proliferative, anti-inflammatory phenotype to a non-proliferative, pro-inflammatory state (Bang et al., 2018). This indicates that GPR37 expression may increase following a toxic insult to promote a pro-resolving response. In fact, the 5’-flanking region of the *GPR37* gene contains a binding site for nuclear factor kappa B, a key inflammatory transcription factor, suggesting that inflammatory stimuli can positively regulate the expression of the receptor. Supporting this hypothesis, we demonstrated that GPR37 expression is upregulated in a macrophage cell line after incubation with the pro-inflammatory mediator lipopolysaccharide (**Figure 1C**). Thus, in a neurodegenerative context, the upregulation of GPR37 observed in PD and AD necropsies could potentially result from infiltrating macrophages involved in the neuroinflammatory response that occurs during disease progression. Alternatively, GPR37 might regulate inflammation through other cell types beyond microglia or macrophages. In the central nervous system, neurons, astrocytes, and oligodendroglia also participate in inflammation. Interestingly, it was found that following ischemic injury, GPR37 expression shifted from mature oligodendrocytes to proliferating oligodendroglia located near the ischemic lesion, where inflammation is expected to occur (Owino et al., 2021). Far from being passive bystanders, recent studies suggest that oligodendroglia actively survey their environment, respond to a wide range of signals, and can perform immunomodulatory functions. Interestingly, it was recently reported that, in response to neurodegeneration, oligodendrocytes react to prosaposin, a putative endogenous agonist of GPR37, secreted by dopaminergic neurons through GPR37-mediated signaling, leading to upregulation of interleukin-6 (Ma et al., 2025). Therefore, the increased GPR37 expression seen in PD and AD necropsies could be linked to immune-related responses, likely originating from infiltrating macrophages or proliferating oligodendroglia responding to inflammatory cues.

Though PD is primarily diagnosed through clinical evaluation, the identification of biomarkers holds significant promise for early detection, tracking disease progression, and distinguishing PD from other neurodegenerative conditions. Biomarkers can be derived from various sources, such as CSF, serum, genetics, and imaging techniques. Among CSF biomarkers, α-synuclein has been the most extensively studied (Yamashita et al., 2023). However, no sustained alterations in α-synuclein levels have been found in PD patients when compared with healthy controls (Morató et al., 2021). Recently, a previous study has shifted focus to the seeding capacity of α-synuclein rather than its total levels, demonstrating its potential for diagnosing early-stage PD (Yamashita et al., 2023). Other CSF biomarkers, including neurofilament, glial fibrillary acidic protein, amyloid beta, and tau, have also been explored in PD, but the findings have been inconsistent or limited to specific PD symptoms (Yamashita et al., 2023). In this context, increased ecto-GPR37 levels have been found in CSF of three independent cohorts of PD patients (Morató et al., 2021; Argerich et al., 2024). While our initial studies did not establish any correlation between ecto-GPR37 levels and clinical ratings (Morató et al., 2021), more recent data suggest that elevated ecto-GPR37 levels are associated with slow disease progression (Argerich et al., 2024). However, there remains a gap in understanding the functional significance of ecto-GPR37 in PD, especially in relation to disease progression. Our hypothesis is that higher GPR37 expression leads to rapid cleavage of the receptor and, consequently, more ecto-GPR37 being shed into the CSF. As shown in **Figure 1**, GPR37 protein levels increase in response to an inflammatory insult. Thus, neuroinflammation in PD may elevate GPR37 expression, leading to increased N-terminal processing and higher levels of ecto-GPR37 in the CSF. Moreover, the reported pro-resolving role of GPR37 counteracting inflammation might explain why ecto-GPR37 is significantly elevated only in patients with slow disease progression. In contrast, those with rapid progression might have less GPR37 expressed, lacking its pro-resolving effects, which could contribute to greater neuroinflammation and a more severe and rapid disease course.

Multiple studies have previously linked PD with elevated inflammatory markers, such as cytokines and interleukins (Yamashita et al., 2023). However, while these markers help distinguish pathological conditions from healthy states, they often lack the specificity needed to differentiate between neurodegenerative diseases. In line with this, when we assessed CSF ecto-GPR37, we observed moderate to high variability among patients with neurodegenerative conditions, thus, no significant differences were found between them. This variability could be due to the stated hypothesis that ecto-GPR37 levels mainly reflect GPR37 upregulation driven by neuroinflammation, a common feature of many neurodegenerative diseases. Furthermore, considering the proposed connection between ecto-GPR37 and inflammation, it is important to account for the potential influence of peripheral inflammatory conditions on ecto-GPR37 levels, especially when assessing these peptides in body fluids other than CSF, such as saliva or serum.

Finally, another key question surrounding ecto-GPR37 peptides is whether they possess any intrinsic biological activity, rather than being inert. Notably, cleaved N-terminal fragments from certain GPCRs, such as protease-activated and adhesion receptors, can function as endogenous ligands, modulating their own receptor conformation and activity through autocrine mechanisms (Peach et al., 2023). Paracrine effects of shed ectodomains have also been documented in various cell surface proteins (Peach et al., 2023), although this remains less explored within the GPCR family. For instance, N-terminal fragments released from adhesion GPCRs such as ADGRB1 and GPR126 can influence neighboring cells independently of their own receptors, with these effects being highly context-dependent, both cellular and pathological (Peach et al., 2023). Establishing an autocrine and/or paracrine function for ecto-GPR37 peptides is particularly relevant given that GPR37 remains an orphan receptor, with no single definitive endogenous ligand identified despite several candidates being proposed. Such a discovery would complete the picture of GPR37 expression and processing, offering critical insights into how it regulates its own activity and modulates surrounding cells. This could significantly broaden our understanding of the role of GPR37 in intercellular communication, particularly in neurodegenerative conditions such as PD.

*We thank Centres de Recerca de Catalunya (CERCA) Programme/Generalitat de Catalunya for IDIBELL institutional support and Maria de Maeztu MDM-2017-0729 to Institut de Neurociencies, Universitat de Barcelona.*

*This work was supported by FEDER/Ministerio de Ciencia, Innovación y Universidades-Agencia Estatal de Investigación (PID2023-147425OB-I00 to FC) and Agència de Gestió d’Ajuts Universitaris i de Recerca (AGAUR) - Generalitat de Catalunya (2021 SGR 00698 to FC).*
